# Immobilization of Captive Kulans (*Equus hemionus kulan*) Without Using Ultrapotent Opioids

**DOI:** 10.3389/fvets.2022.885317

**Published:** 2022-08-10

**Authors:** Julia Bohner, Johanna Painer, Denyse Bakker, Anna Jean Haw, Hanna Rauch, Eva Maria Greunz, Beate Egner, Frank Goeritz

**Affiliations:** ^1^Leibniz Institute for Zoo and Wildlife Research (IZW), Berlin, Germany; ^2^Serengeti-Park Department of Research, Hodenhagen, Germany; ^3^Department of Integrative Biology and Evolution (FIWI), University of Veterinary Medicine Vienna, Vienna, Austria; ^4^Lammermoor Veterinary Clinic, Krugersdorp, South Africa; ^5^Brain Function Research Group, Faculty of Health Sciences, School of Physiology, University of the Witwatersrand, Johannesburg, South Africa; ^6^Center of Zoo and Wild Animal Health, Copenhagen Zoo, Frederiksberg, Denmark; ^7^Veterinary Academy of Higher Learning (VAHL), Babenhausen, Germany

**Keywords:** zoological medicine, wildlife, kulan, immobilization, equine, general anesthesia, etorphine, alpha-2 agonist

## Abstract

Etorphine is widely used in zoological medicine for the immobilization of large herbivores. All reported immobilization protocols for kulans use etorphine as the primary immobilizing agent. However, etorphine can trigger severe side effects and is highly toxic for humans, its availability is occasionally limited for use in wildlife medicine. Therefore, two different alpha-2 agonist-based protocols for the general anesthesia of kulans were investigated and compared with the standard etorphine immobilization. In total, 21 immobilizations were performed within the scope of routine husbandry management at the Serengeti-Park Hodenhagen. Kulans were darted using a ketamine–medetomidine–midazolam–butorphanol (KMMB) protocol (*n* = 8, treatment group (TG) 1), a tiletamine–zolazepam–medetomidine–butorphanol (TZMB) protocol (*n* = 7, treatment group (TG) 2), or an etorphine–acepromazine–detomidine–butorphanol (EADB) protocol (*n* = 6, control group). Vital parameters included heart rate, respiratory rate, arterial blood pressure (invasive), end tidal CO_2_ (etCO_2_), electromyography and core body temperature, which were all assessed every 10 min. For blood gas analysis, arterial samples were collected 15, 30, 45 and 60 min after induction. Subjective measures of quality and efficacy included quality of induction, immobilization, and recovery. Time to recumbency was longer for TG 1 (9.00 ± 1.67 min) and TG 2 (10.43 ± 1.79 min) compared to the induction times in the control group (5.33 ± 1.93 min). Treatment group protocols resulted in excellent muscle relaxation, normoxemia and normocapnia. Lower pulse rates combined with systolic arterial hypertension were detected in the alpha-2 agonist-based protocols. However, only in TZMB-immobilized kulans, sustained severe systolic arterial hypertension was observed, with significantly higher values than in the TG 1 and the normotensive control group. At 60 min following induction, medetomidine and detomidine were antagonized with atipamezole IM (5 mg/mg medetomidine or 2 mg/mg detomidine), etorphine and butorphanol with naltrexone IV (2 mg/mg butorphanol or 50 mg/mg etorphine), and midazolam and zolazepam with flumazenil IV (0.3 mg per animal). All three combinations provided smooth and rapid recoveries. To conclude, the investigated treatment protocols (KMMB and TZMB) provided a safe and efficient general anesthesia in kulans with significantly better muscle relaxation, higher respiration rates and improved arterial oxygenation compared with the immobilizations of the control group. However, the control group (EADB) showed faster recoveries. Therefore, EADB is recommended for ultra-short immobilizations (e.g., microchipping and collaring), especially with free-ranging kulans where individual recovery is uncertain, whereas the investigated treatment protocols are recommended for prolonged medical procedures on captive kulans.

## Introduction

Kulans (*Equus hemionus kulan*) are a subspecies of the Asiatic wild ass. Anesthesia in kulans can be particularly challenging due to their stoic nature (1). Adaptation to the desert influences fluid and electrolyte balance and accordingly, modifies the metabolism of certain pharmacological agents (2). These physiological alterations also affect pharmacokinetic and pharmacodynamic characteristics of immobilizing agents; hence, increased dosages of anesthetics may be required (3).

The ultrapotent opioid etorphine is the most commonly used agent for the remote immobilization of wild equids (4–12). Small volumes of etorphine can be lethal for humans, and therefore, it is subject to special purchase regulations. Consequently, availability of etorphine in certain countries is sometimes limited, and reliable alternatives are needed (7, 13, 14).

Etorphine, in combination with low doses of detomidine, acepromazine and butorphanol, has been used to successfully capture free-ranging kulans (9, 11). Tachycardia, hypertension, hypoxemia, hypercapnia, metabolic acidosis, muscle rigidity and tremors are common side effects and numerous studies have documented the cardiorespiratory effects of etorphine (7–9, 11, 15, 16). However, the limited availability of etorphine, as well as its high toxicity for humans, has encouraged veterinarians to test other combinations (7). For wild equids, there is only a limited number of studies of anesthesia combinations without ultrapotent opioids (7, 13, 14, 17–20). The combination of medetomidine and ketamine (MK) has been successfully used for remote anesthesia of non-domestic equids (13, 14, 18, 20). Potential side effects of these combinations include ataxia during induction and recovery, bradycardia, hypoxemia and hypertension. However, in some cases, MK failed to induce recumbency and was therefore used rarely (13, 18). A combination of ketamine, medetomidine and butorphanol (KMB) was recommended for routine management procedures of captive zebras (7). This anesthetic regime provided an effective immobilization characterized by a deep plane of anesthesia and excellent muscle relaxation. Side effects of KMB include severe systemic hypertension and moderate hypoxemia. No etorphine-free anesthesia protocols have been reported for kulans.

Recent studies on the white rhinoceros focussed on the attenuating influence of midazolam on peripheral oxygen consumption (21). It has been shown that the excellent muscle relaxation properties of midazolam ensures deep ventilation and thus improves peripheral tissue oxygenation in rhinos (22). Furthermore, midazolam may lower the dosage of medetomidine, and reduce alpha-2 agonist-mediated vascular hypertension (23). We therefore hypothesized that adding midazolam to the KMB protocol may have beneficial effects on cardiorespiratory parameters, such as oxygenation and blood pressure in kulans.

All etorphine-free drug combinations with commercially available ketamine, medetomidine, midazolam and butorphanol (KMMB) resulted in a large volume for darting which potentially limits its use in free-ranging settings (17, 18, 20). Furthermore, the poor solubility of concentrated ketamine preparations also restricts the use of higher concentrations of this drug in small volumes (13). Tiletamine has greater solubility than ketamine (24, 25), which facilitates the application of 3cc darts for remote drug administration. Tiletamine is commercially available as a dry substance in combination with zolazepam (Zoletil® or Telazol®), a benzodiazepine-like tranquilizer (26). The combination of tiletamine, zolazepam and medetomidine (TZM) has been successfully used for distance immobilization of feral horses, where it induces adequate, safe and partially reversible anesthesia (17). With the advantage of high solubility, in the current study, tiletamine and zolazepam were added to the combination of medetomidine and butorphanol (TZMB), in order to provide a highly concentrated alternative to KMMB, which could also be used in free-ranging settings.

This study aims to establish and to evaluate two safe and effective alpha-2 agonist-based protocols for captive kulans and to compare the results with the currently used etorphine-based drug combination (the prevailing gold standard). The cardiorespiratory parameters and benefits of both combinations were determined. As far as the authors are aware, this is the first report on these drug combinations in non-domestic equids. We hypothesize less respiratory depression, better induction quality and muscle relaxation, for the two etorphine-free combinations.

## Materials and Methods

### Study Approval

This randomized, prospective study was approved by the Committee for Ethics and Animal Welfare of the Leibniz-IZW (approval no: 2017-02-03).

### Animals and Housing

All immobilizations (*n* = 21) were performed on healthy captive kulans (*n* = 18) for animal management reasons. These included genetic assessments, general health checks, hoof trimmings, dental treatments, screening for infectious diseases and vaccinations. A group of 14 females and four males were included in the study; three kulans had to be anesthetized a second time for reproductive management reasons and transportation. The animals were kept under extensive holding conditions in the four-hectare-sized drive-through enclosure of the Serengeti-Park Hodenhagen, Germany (52°44′54.8“N 9°37′14.4″E/elevation: 24 m above sea level). For these interventions, the kulans were temporary housed in a confined area with three compartments (each 17 ×17 m), divided by sliding doors to allow individual separation for darting, as well as safe and rapid access to the immobilized animal. The fences of the enclosure were covered with canvas, to reduce stress. Kulans were darted and observed from an elevated position. Prior to darting, all animals had *ad libitum* access to hay and water and underwent 3 days of habituation to the new enclosure.

### Drug Combinations

Prior to immobilization of each animal, the bodyweight was estimated based on exterior body condition and drug dosages were calculated accordingly.

Three drug combinations were compared:

- KMMB (treatment group (TG 1, *n* = 8 anesthesia): 3 mg/kg ketamine hydrochloride (Ketamidor 100 mg/mL, Richter Pharma AG, Wels, Austria), 0.09 mg/kg medetomidine hydrochloride (medetomidine 40 mg/mL, Wildlife Pharmaceuticals Ltd., White River, Mpumalanga, South Africa), 0.3 mg/kg midazolam hydrochloride (midazolam 50 mg/mL, University of Vienna, Vienna, Austria) and 0.2 mg/kg butorphanol tartrate (butorphanol 50 mg/mL, Wildlife Pharmaceuticals Ltd., White River, Mpumalanga, South Africa).
- TZMB (TG 2, *n* = 7 anesthesia): 250 mg/animal tiletamine and 250 mg/animal zolazepam (Zoletil®100, Virbac, Glattbrugg, Switzerland), 0.09 mg/kg medetomidine (Medetomidine 40 mg/mL, Wildlife Pharmaceuticals Ltd., White River, Mpumalanga, South Africa) and 0.2 mg/kg butorphanol (Butorphanol 50 mg/mL, Wildlife Pharmaceuticals Ltd., White River, Mpumalanga, South Africa).
- EADB (control group, *n* = 6 immobilizations) was defined as the control group, using the same dosage as used by Walzer et al. (11) with one dosage for every similarly sized adult individual: 2.45 mg/animal etorphine hydrochloride (M99 R 9.8 mg/mL, Novartis South Africa (Pty) Ltd, Midrand, South Africa), 10 mg/animal acepromazine maleate (Vetranquil 1%, Ceva Tiergesundheit, Düsseldorf, Germany), 10 mg/animal butorphanol tartrate (Butorgesic 10 mg/mL, CP-Pharma Handelsgesellschaft mbH, Burgdorf, Germany) and 10 mg/animal detomidine hydrochloride (Cepesedan 10 mg/mL, CP-Pharma Handelsgesellschaft mbH, Burgdorf, Germany).

For immobilization, one animal was separated in an empty compartment and darted with a gas-powered rifle (Daninject CO_2_ Injection Rifle Model JM.DB.13, DAN-Inject Smith GmbH, Walsrode, Germany) using a 3cc dart equipped with a collared needle (20 x 40-mm collared dart needle, DAN-Inject Smith GmbH, Walsrode, Germany). All combinations were administered intramuscularly (IM, gluteal, trapezius or triceps brachii muscle) from a distance of approximately 14 to 16 m.

### Quantitative and Qualitative Assessment of Immobilization

Induction time was defined as the time from drug administration (full dose) to recumbency. The time of recumbency was recorded as timepoint zero (TP0). The time from TP0 to antidote administration was defined as the immobilization time. Recovery time was defined as the time from antidote administration to standing. The quality of induction was based on a subjective descriptive scale [SDS; adapted from Stemmet et al. (7)] and rated as excellent (1), good (2), fair (3), or poor (4). The quality of immobilization was assessed according to the maintenance of reflexes (eyelid, rectal sphincter) and a subjective immobilization quality score (1 = limited effect, 2 = deep sedation, 3 = light immobilization plane, 4 = deep immobilization plane, 5 = excessively deep). Qualitative assessment of recovery from anesthesia was rated as excellent (1), good (2), fair (3), or poor (4) using an SDS described by Stemmet et al. (7).

### Monitoring

Once the animal was in lateral recumbency, eyedrops (Hylo gel Ursapharm Arzneimittel GmbH, Saarbrücken, Germany), a blindfold and earplugs were applied. Thereafter the animals were placed on a carrier sheet and moved to an adjacent examination room where the actual body weight was measured (Kranwaage, 3.000/1 kg, LED, Steinberg Systems, Germany). The kulans were placed on a table in left-sided lateral recumbency for further assesment. Monitoring of physiological parameters included respiratory rate (RR), heart rate (HR), oxygen saturation (SpO_2_) and rectal body temperature (RT). Peripheral oxygen hemoglobin saturation (SpO_2_) and heart rate (HR) were measured using a reflectance pulse oximeter probe (Masimo Rad-57 Veterinary Pulse Oximeter, Masimo Europe Ltd., Puchheim, Germany) attached to the tongue. The respiratory rate was determined by visual observation of chest movements, and the rectal temperature was obtained using a rectal thermometer (RT) (Dormoterm rapid Fieberthermometer, Uebe Medical GmbH, Kühlsheim, Germany). All monitoring parameters were assessed continuously by the same person and recorded every 10 minutes from TP0 to TP60 throughout the immobilization period.

For blood gas analysis (CG4+ cartridges, ISTAT, Abaxis Europe GmbH, Griesheim, Germany), the first arterial sample (TP0) was collected anaerobically from the carotid artery using a 1-mL pre-heparinised syringe (Kabevette, Kabe Labortechnik GmbH, Nümbrecht, Germany) equipped with a 22-gauge needle (Microlance™ 22G-0,7 × 30 mm–schwarz, BD, Heidelberg, Germany). Spontaneous filling of the syringe confirmed arterial sampling. After collection of the arterial sample, the syringe was sealed with a lid to prevent air contamination. The aseptic placement of a 22-gauge catheter (Vasovet Braunüle; B Braun Vetcare GmbH, Tuttlingen, Germany) in the right facial artery facilitated collection of subsequent arterial samples (TP 15, 30, 45) and was also used for continuous monitoring of the arterial blood pressure. Immediate evaluation (point of care) of oxygen saturation (SaO_2_), partial pressure of oxygen (PaO_2_), partial pressure of carbon dioxide (PaCO_2_) and acid–base state, such as pH and lactate levels, were performed using an ISTAT portable analysis device. Based on the formula F_I_O_2_ (P_B_ – P_H2O_) – (PaCO_2_/RQ) – PaO_2_ (PaO_2_ = arterial partial pressure of oxygen; PaCO_2_ = arterial partial pressure of carbon dioxide) the alveolar-arterial oxygen gradient [P(A-a)O_2_] (mmHg) was calculated. P_B_ the barometric pressure and PH2O the partial pressure of water vapor were calculated as (P_B_) = 760 ^*^ e(altitude above sea level/-7000) mmHg and as PH2O = 4.58 EXP{(17.27 × Tb)/(237.3 + Tb)} mmHg, where Tb is the body temperature. RQ (respiratory quotient) was assumed to be 0.9, the norm in conscious healthy horses (26), and the inspired oxygen fraction was calculated with F_I_O_2_ = 20% + (4 x oxygen flow).

A nasal tube (endotracheal tube 11.0 mm x 15.3 mm x 38 cm; WDT, Garbsen, Germany) was placed in the ventral meatus of the right nostril and attached to a portable side stream capnograph measuring RR and end tidal carbon dioxide partial pressure (etCO_2_) (EMMA; Masimo Europe Ltd, Puchheim, Germany). A 14-gauge catheter (Vasovet Braunüle; B Braun Vetcare GmbH, Tuttlingen, Germany) was aseptically placed in the left jugular vein.

A portable patient-side arterial pressure monitor (IntraTorr; IntraVitals, UK) continuously measured invasive systolic arterial blood pressure (SAP), diastolic arterial blood pressure (DAP) and mean arterial blood pressure (MAP), as well as pulse rate (PR). This monitor and a non-compliant tubing (Fluid line; IntraVitals, UK) were connected to an electronic strain gauge transducer (Deltran II transducer; IntraVitals, UK). Prior to each initial measurement, these were zeroed to the atmospheric pressure at the level of the right atrium.

The forehead of each kulan was clipped, ultrasound gel was applied, and electrodes were attached to measure myoelectrical activity (EMG) using a Masimo Root (Masimo Austria GmbH, Vienna, Austria).

During the immobilization, oxygen was supplied at a flow rate of 3 L/minute *via* a small tube in the left nostril, after the first arterial sample was taken at TP10. Each animal received intravenous isotonic sodium chloride infusion (NaCl 0.9 %, B Braun Vetcare GmbH, Tuttlingen, Germany) with a drip rate of 10 mL/kg/h as supportive care.

### Data Analysis

Statistical analyses were performed by using the R software package for Mac OS X (27). No signs of non-linearity or heteroscedasticity were detected. A linear mixed-effect model was therefore used for repeated measures (28) to analyse the physiological parameters (RR, HR, SpO_2_, RT, SAP, DAP, MAP, NIRS, EMG and etCO_2_) and the blood gas variables (SaO_2_, PaO_2_, PaCO_2_, pH, BEecf, HCO_3_ and TCO_2_). The assumptions of the model, in particular normal distribution and homoscedasticity, were checked by creating a plot and a histogram of the model residuals.

Immobilization time (minutes) and treatment vs. the control group (immobilization protocol) were used as independent variables with a two-way interaction term between these predictors. The subject was included as a random factor in each model to correct for individual differences in treatment groups. Changes in physiological parameters and blood gas samples over time, as well as between treatment groups and the control group (e.g., EADB vs. TZMB or KMMB) were analyzed using a *post hoc* test. The predicted means and standard error of means (SEM), calculated for each variable over time and between groups, were finally plotted (29).

In addition, a one-way analysis of variance (ANOVA) following a *post hoc* test (30) was performed to test whether the recovery time differed between the treatment groups. To determine if there were statistically significant differences and to test whether there was a variety between the scoring quality (ordinal variable) and treatment groups (categorical variable), a Kruskal–Wallis rank sum test was applied. Pairwise multiple comparisons were calculated using a pairwise Wilcoxon rank sum test (function pairwise.wilcox.test) to identify which groups were different. When statistically significant differences were observed, a Wilcoxon test (Bonferroni) was used to adjust for multiple testing.

Finally, to identify a linear relationship between EMG and the subjective muscle relaxation score, a linear model was fitted. A *p* < 0.05 was considered statistically significant.

## Results

### Drug Dosages

The kulans had a mean bodyweight (BW) of 166.67 ± 37.44 kg (mean ± standard error, range: 101.0–224.0 kg). The dosages (± standard deviation/SD) calculated on the actual body weight were efficient enough to cause a reliable immobilization in kulans, without supplementary immobilizing agents (Table 1).

**Table 1:** Drug dosages for induction and reversal of immobilization, in TG^a^ 1: ketamine–medetomidine–midazolam–butorphanol (KMMB), TG 2: tiletamine–zolazepam–medetomidine–butorphanol and CG^b^: etorphine–acepromazine–detomidine–butorphanol (EADB) immobilized kulans (Equus hemionus kulan).

**Induction protocol**					
**TG 1**	**Dosage mg/kg**	**TG 2**	**Dosage mg/kg**	**CG**	**Dosage mg/kg**
ketamine	3.69 ± 0.69	tiletamine	1.36 ± 0.23	etorphine	0.02 ± 0, 01
medetomidine	0.1 ± 0.02	zolazepam	1.36 ± 0.23	acepromazine	0.06 ± 0, 02
midazolam	0.34 ± 0.07	medetomidine	0.08 ± 0.02	butorphanol	0.06 ± 0, 01
butorphanol	0.25 ± 0.11	butorphanol	0.19 ± 0.04	detomidine	0.06 ± 0, 01
**Recovery protocol**					
**TG 1**	**Ratio**	**TG 2**	**Ratio**	**CG**	**Ratio**
A/M^d^	5 mg/mg IM^e^	A/M	5 mg/mg IM	A/D	2 mg/mg IV^f^
N/B^g^	2 mg/mg IV	N/B	2 mg/mg IV	N/B	50 mg/mg IV
F^h^	0.3 mg IV	F	0.3 mg IV	na^i^	na

*Values are reported as mean (± SD^c^ range). Dosages for antagonism are also given*.

a *TG, treatment group*;

b *CG, control group*;

c *SD, standard deviation*;

d *A/M, atipamezole/medetomidine*;

e *IM, intramuscular*;

f *IV, intravenously*;

g *N/B, naltrexone/butorphanol*;

h *F, flumazenil*;

i *na, not applicable; missing values / no measurements available*.

### Quality of Immobilization

Induction times were longer for the KMMB (9.00 ± 1.67 min) and TZMB (10.43 ± 1.79 min) treatment groups than for the control group (5.33 ± 1.93 min) (Table 1). Anesthetic induction in kulans with KMMB and TZMB was characterized by the typical signs of a standing sedation, such as standing still with a wide based stance, head down, and a relaxed jaw with bottom lip hanging. The transition to recumbency was initiated with mild to severe swaying. In best cases, the kulans fell over into sternal recumbency with an elevated head position, followed by a smooth transition to lateral recumbency. In worst cases, the kulans resisted the transition to recumbency for a longer period. These cases revealed sequences of ataxia, stumbling and falling repeatedly. Mild tremors were only observed in two TZMB kulans (KMMB and TZMB median scores = 2/good). Immobilization of the kulans induced with EADB was characterized by a strong tendency to move forward with the typical hypermetric “hackneyed” gait and stargazing. Consequently, several kulans walked into the fencing, where they exhibited head pressing and pacing on the spot. The transition to recumbency was typically proceeded by an initial contracting of the hind legs and an elevated head. All animals of the control group showed increased jaw tension, involuntary leg movements, and moderate to severe tremors and cramps following recumbency. Within the EADB group, two kulans with severe seizures and spontaneous leg movements following recumbency required 10 mg of midazolam IV to reduce tremor and seizures, as well as two doses of 100 mg ketamine IV, to reach full recumbency; three other control animals required 10 mg midazolam IV, to stop the cramping (control group median score = 4/poor). The induction quality scores are summarized in (Table 2).

**Table 2:** Distribution of scores that represent the quality of induction in TG^a^ 1: ketamine–medetomidine–midazolam–butorphanol (KMMB), TG 2: tiletamine–zolazepam–medetomidine–butorphanol and CG^b^: etorphine–acepromazine–detomidine–butorphanol (EADB) immobilized kulans (Equus hemionus kulan).

**Induction quality scores**	**1**	**2**	**3**	**4**	
**Description**	**Excellent**	**Good**	**Fair**	**Poor**	
**Attempts to stand**	<2	> 2	numerous	Not staying in recumbency, darting again or hand injection	
**Level of ataxia/ tremors**	Slight to none	Moderate	Severe	Very severe	
**Group**	**Number of animals**				**Scores**
**TG 1**	2	6	0	0	2 ± 0.164
**TG 2**	3	3	1	0	2 ± 0.286
**Control**	0	0	1	5	4 ± 0.167

*Values are reported as median (± SE^c^ range)*.

a *TG, treatment group*;

b *CG, control group*;

c *SE, standard error*.

Over the course of immobilization all animals maintained palpebral and anal reflexes. KMMB and TZMB kulans showed an excellent muscle relaxation and did not require any additional anesthetics. By contrast, the control group exhibited increased muscle tension, involuntary leg movements, tremors and, sustained seizures. *n* = 1 kulan showed tremors and spontaneous leg movements during recumbency at TP 45. Additional administration of 2 ×5 mg midazolam only slightly resolved these side effects. At TP 50, the same kulan attempted to rise and hence received 2 times 100 mg ketamine to maintain recumbency. Scoring of muscle relaxation and consequently the immobilization quality for both treatment groups revealed a median of 3 (light to moderate immobilization level), while the control group showed a median of 2 (deep sedation). All these results are summarized as immobilization quality scores (Table 3).

**Table 3:** Distribution of scores representing the quality of immobilisation in TG^a^ 1: ketamine–medetomidine–midazolam–butorphanol (KMMB), TG 2: tiletamine–zolazepam–medetomidine–butorphanol and CG^b^: etorphine–acepromazine–detomidine–butorphanol (EADB) immobilized kulans (Equus hemionus kulan).

**Immobilization scores**	**1**	**2**	**3**	**4**	**5**	
**Description**	**Limited effect**	**Deep sedation**	**Light immobilization**	**Deep immobilization**	**Excessively deep**	
**Recumbency**	No	Yes	Yes	Yes	Yes	
**Muscle activity**	No recumbency acchieved	Spontaneous and/ or tremors	Reduced to complete relaxation	Complete relaxation	Too deep	
**Palpebral and anal reflex**	Intact and vigorous	Intact and vigorous	Intact but sluggisch	Very sluggish to absent	Absent	
**Group**	**Number of animals**					**Scores**
**TG 1**	n = 0	n = 0	n = 5	n = 3	n = 0	3 ± 0.162
**TG 2**	n = 0	n = 0	n = 4	n = 3	n = 0	3 ± 0.171
**Control**	n = 1	n = 5	n = 0	n = 0	n = 0	2 ± 0.289

*Values are reported as median (± SE^c^ range)*.

a *TG, treatment group*;

b *CG, control group*;

c *SE, standard error*.

Recovery times were longer for KMMB (6.00 ± 1.10 min) and TZMB (6.29 ± 1.18 min) treatment groups than for the control group (3.50 ± 1.27 min) (Table 1). The recovery quality was excellent in all groups, with a median score of 1 (Table 4). Final positioning of the kulans in sternal recumbency and leaving them alone following antidote administration resulted in calm and smooth transitions from sternal positions to standing. Most kulans remained temporarily in sternal recumbency with their heads up, until they were able to stand up in a controlled way, often at the first attempt; two kulans of the TZMB protocol rolled to lateral recumbency before getting up. Treatment group kulans showed a slight ataxia and swaying while walking away with a protruding tongue, while control group kulans showed almost no signs of ataxia.

**Table 4:** Distribution of scores representing the quality of recovery in TG^a^ 1: ketamin–medetomidin–midazolam–butorphanol (KMMB), TG 2: tiletamine–zolazepam–medetomidin–butorphanol and CG^b^: etorphine–acepromazine–detomidin–butorphanol (EADB) immobilized kulans (Equus hemionus kulan).

**Recovery quality scores**	**1**	**2**	**3**	**4**	
**Description**	**Excellent**	**Good**	**Fair**	**Poor**	
**Attempts to stand**	<2	> 2	numerous	Not staying in recumbency, darting again or handinjection	
**Level of ataxia/ tremors**	Slight to none	Moderate	Severe	None, requires clinical examination	
**Group**	**Number of animals**				**Scores**
**TG 1**	6	2	0	0	1 ± 0.164
**TG 2**	6	1	0	0	1 ± 0.143
**Control**	6	0	0	0	1 ± 0^*^^†^^d^

*Values are reported as median (± SE^c^ range)*.

a *TG, treatment group*;

b *CG, control group*;

c *SE, standard error*;

c *na, not applicable: missing values as no measurements available*;

d * *Significantly different to KMMB at this timepoint (p < 0.05)*;

† *Significantly different to TZMB at this timepoint (p < 0.05)*.

### Monitoring Data

All the vital parameters are summarized in Table 5. The EMG values were significantly lower in the TZMB group (*p* < 0.02) and from TP20 to TP60 in the KMMB group (*p* < 0.001) than in the control group. Only at TP20, the EMG values were significantly lower in the TZMB group than in the KMMB group (*p* < 0.0013), while there were no significant differences between the treatment groups from TP30 to TP60 (Table 5). Activated EMG was attributed to increased muscle contraction. Hence, improved muscular relaxation was associated with low EMG activities.

**Table 5:** Vital parameters for TG^a^ 1: ketamine–medetomidine–midazolam–butorphanol (KMMB), TG 2: tiletamine–zolazepam–medetomidine–butorphanol (TZMB), and CG^b^: etorphine–acepromazine–detomidine–butorphanol (EADB), immobilized kulans (Equus hemionus kulan) following recumbency at timepoint 0 for a 60-minute period.

**Parameter^d^**	**Protocol**	**TP^e^ 0**	**TP 10**	**TP 20**	**TP 30**	**TP 40**	**TP 50**	**TP 60**
**RR (breaths per minute**)	**TG 1**	23 ± 2.49	15 ± 2.49	16 ± 2.49^†^^f^	18 ± 2.40	16 ± 2.49	18 ± 2.90	23 ± 3.51
	**TG 2**	25 ± 4.20	20 ± 3.45	20 ± 2.83	17 ± 2.87	22 ± 2.66	21 ± 2.82	19 ± 3.19
	**Control**	14 ± 5.91	15 ± 3.53	14 ± 3.28	14 ± 2.76	16 ± 3.22	12 ± 3.17^†^	18 ± 3.53
**Temp (** **°** **C)**	**TG 1**	39.0 ± 0.28	Na^g^ ± na	38.4 ± 0.37	38.3 ± 0.28	37.8 ± 0.43	37.9 ± 0.55	na ± na
	**TG 2**	38.9 ± 0.28	39.2 ± 0.40	38.0 ± 0.32	37.7 ± 0.36	38.0 ± 0.30	37.6 ± 0.30	37.4 ± 0.33
	**Control**	40.1 ± 0.30	na ± na	38.3 ± 0.57	38.7 ± 0.34	39.2 ± 0.37	39.1 ± 0.53	na ± na
**SpO**_**2**_ **(%)**	**TG 1**	96.0 ± 1.54	96.0 ± 1.31	96.7 ± 1.24	96.2 ± 1.18	96.7 ± 1.18	95.4 ± 1.31	95.8 ± 2.08
	**TG 2**	na ± na	95.5 ± 1.27	97.2 ± 1.27	96.8 ± 1.39	96.7 ± 1.30	96.5 ± 1.37	96.9 ± 1.55
	**Control**	86.1 ± 1.32^*^	89.2 ± 1.42^*^^†^	94.2 ± 1.58	94.6 ± 1.32	94.0 ± 1.43	89.3 ± 1.55	93.6 ± 1.74
**etCO**_**2**_ **(mmHg)**	**TG 1**	56 ± 4.36	41 ± 2.81	42 ± 2.70	45 ± 2.70	42 ± 2.70	44 ± 3.11	43 ± 3.72
	**TG 2**	56 ± 5.86	45 ± 3.02	41 ± 3.01	42 ± 3.07	44 ± 3.07	46 ± 3.00	44 ± 3.41
	**Control**	na ± na	45 ± 4.47	42 ± 3.52	45 ± 2.99	46 ± 3.45	51 ± 3.82	49 ± 4.46
**EMG (mV)**	**TG 1**	na ± na	na ± na	28.6 ± 7.9^†^	10.9 ± 6.69	5.0 ± 5.96	4.7 ± 6.66	3.5 ± 6.68
	**TG 2**	na ± na	−0.2 ± 7.62	1.6 ± 5.59	1.1 ± 4.71	1.5 ± 5.08	2.3 ± 4.69	1.6 ± 5.59
	**Control**	na ± na	na ± na	76.9 ± 10.43^*^^†^	58.9 ± 10.43^*^^†^	62.8 ± 7.82^*^^†^	68.7 ± 7.82^*^^†^	33.8 ± 6.45^*^^†^

*Parameters are reported as mean (± SE^c^; range) at 10-minute intervals*.

a *TG, treatment group*;

b *CG, control group*;

c *SE, standard error*;

d *RR, respiration rate; Temp, rectal temperature; SpO_2_, peripheral oxygen haemoglobin saturation; etCO_2_, end tidal carbon dioxide; EMG, electromyography*;

e *TP, timepoint*;

f * *Significantly different to KMMB at this timepoint (p < 0.05)*;

† *Significantly different to TZMB at this timepoint (p < 0.05)*;

g *na, not applicable: missing values / no measurements available*.

**Figure 1:**
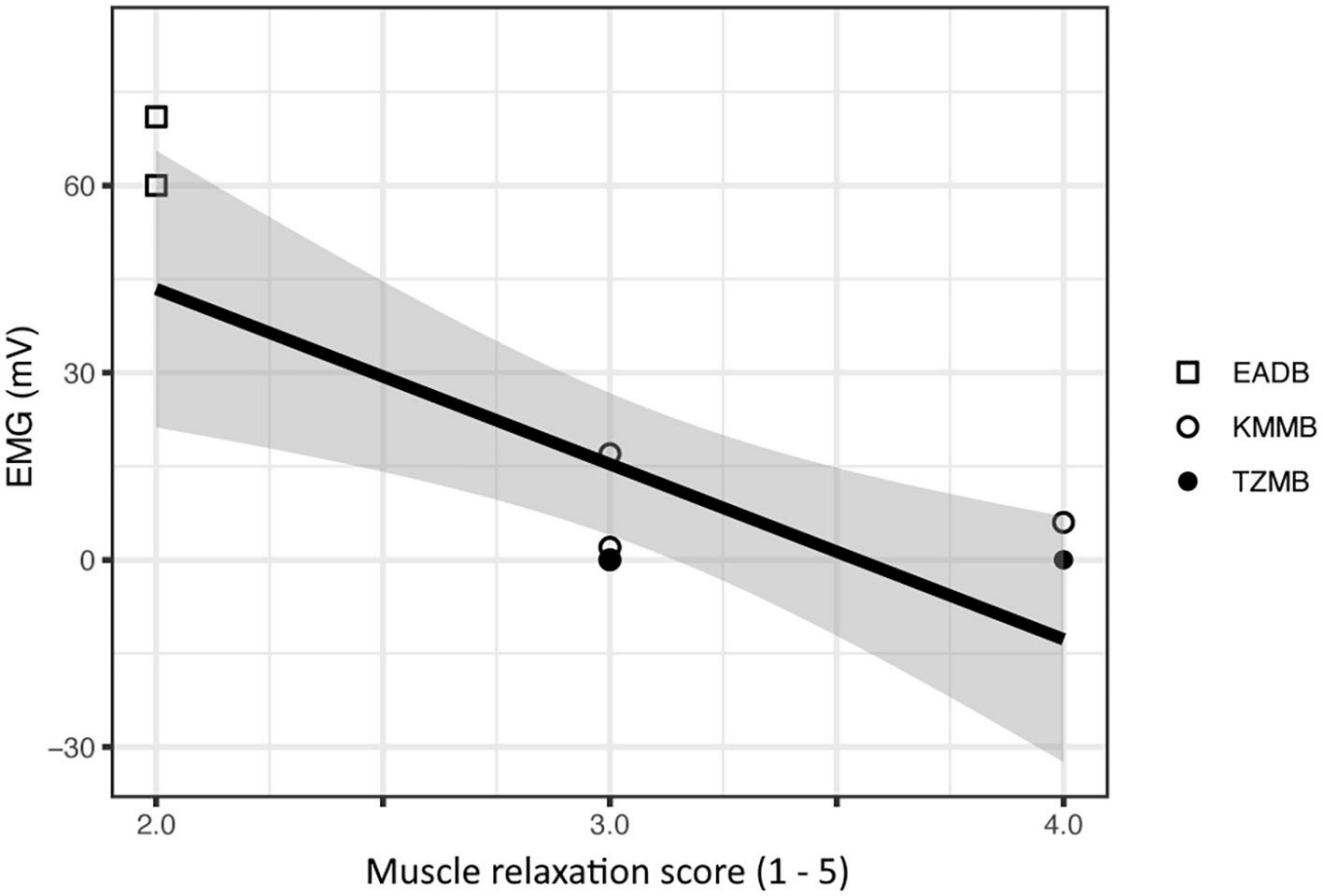
Comparison of subjective muscle relaxation scoring and electromyography (EMG) measurements for treatment group 1 (KMMB), treatment group 2 (TZMB) and the control group (EADB). The figure presents a significant negative correlation where higher EMG values seen in the control group show low subjective muscle relaxation scores and subsequently lower EMG values that correspond to a higher subjective muscle relaxation score in the treatment groups.

There was a significant negative relationship between the EMG measurements and the subjective muscle relaxation score (*p* < 0.001) (Figure 1).

**Figure 2:**
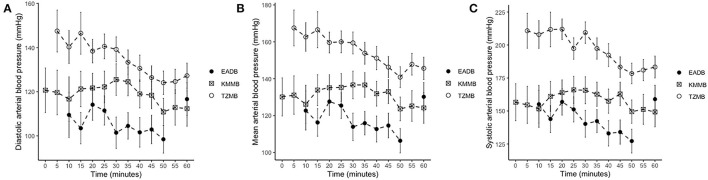
Illustration of invasive blood pressure (IBP) over time. Values of each timepoint are the means for treatment group 1 (KMMB), treatment group 2 (TZMB) and the control group (EADB) ± standard error (error bars). **(A)** Comparison of systolic arterial IBP between TG 1, TG 2 and control group. **(B)** Comparison of mean arterial IBP between TG 1, TG 2 and control group. **(C)** Comparison of diastolic arterial IBP between TG 1, TG 2 and control group.

IBP measurements revealed higher SAP, MAP and DAP values for the treatment groups (Table 6, Figures 2A–C). Only in TZMB-immobilized kulans, severe systemic hypertension was observed with significantly higher values than in TG 1 and the normotensive control group. By contrast, HR in the EADB kulans revealed higher values for each timepoint than the treatment groups (*p* < 0.001) (Table 6). RR and etCO_2_ did not differ significantly between treatment groups. It should be taken into consideration that etCO2 values measured with a capnograph attached to a nasal tube may differ from etCO2 values of endotracheally intubated animals (Table 5). Both treatment groups revealed normoxemia (SpO_2_ > 95%) over the course of anesthesia with significantly higher SpO_2_ values during the first 10 min of immobilization, before oxygen was supplemented at TP10, than the hypoxemic control group (8). Furthermore, both treatment groups were hyperthermic at TP0 and returned to normothermia from TP20 to TP60, while the control group remained hyperthermic over the course of immobilization. At TP0 and TP40, both treatment groups revealed a significantly lower temperature than the control group (Table 5).

**Table 6:** Vital parameters of blood pressure and heartrate for TG^a^ 1: ketamine–medetomidine–midazolam–butorphanol (KMMB), TG 2 tiletamine–zolazepam–medetomidine–butorphanol (TZMB), and CG: etorphine–acepromazine–detomidine–butorphanol (EADB) immobilized kulans (Equus hemionus kulan) following recumbency at timepoint 0 for a 60-minute period.

**Parameter^d^**	**Protocol**	**TP^e^ 0**	**TP 10**	**TP 20**	**TP 30**	**TP 40**	**TP 50**	**TP 60**
**sIBP (mmHg)**	**TG 1**	157 ± 14.04	152 ± 14.04^†^^f^	164 ± 9.66^†^	166 ± 10.25^†^	157 ± 9.18^†^	150 ± 8.87^†^	149 ± 11.33^†^
	**TG 2**	na^g^ ± na	208 ± 10.53	212 ± 7.82	209 ± 8.17	192 ± 8.61	178 ± 7.80	183 ± 8.28
	**Control**	na ± na	155 ± 14.25^†^	157 ± 10.13^†^	140 ± 10.13^†^	133 ± 9.59^†^	127 ± 8.99^†^	159 ± 10.53
**mIBP (mmHg)**	**TG 1**	130 ± 10.39	126 ± 10.39^†^	135 ± 7.06^†^	137 ± 7.51^†^	132 ± 6.70^†^	124 ± 6.46^†^	124 ± 8.34
	**TG 2**	na ± na	163 ± 7.75	160 ± 5.68	159 ± 5.95	151 ± 6.29	141 ± 5.66	146 ± 6.03
	**Control**	na ± na	123 ± 10.55^†^	128 ± 7.42^†^	114 ± 7.42^†^	113 ± 7.02^†^	106 ± 6.56^†^	130 ± 7.74
**dIBP (mmHg)**	**TG 1**	121 ± 10.12	117 ± 10.12	122 ± 6.71	126 ± 7.18	119 ± 6.36	111 ± 6.11	112 ± 8.03
	**TG 2**	na ± na	140 ± 7.48	138 ± 5.33	139 ± 5.62	139 ± 5.62	124 ± 5.32	127 ± 5.69
	**Control**	na ± na	110 ± 10.28^†^	114 ± 10.12^†^	102 ± 7.10^*^^†^	102 ± 6.71^†^	99 ± 6.23^†^	117 ± 7.46
**Heartrate (BPM)**	**TG 1**	44 ± 3.41	41 ± 3.41	40 ± 3.31	40 ± 3.31	43 ± 3.31	41 ± 3.41	40 ± 3.71
	**TG 2**	39 ± 4.28	41 ± 3.41	41 ± 3.26	38 ± 3.44	41 ± 3.41	41 ± 3.26	40 ± 4.02
	**Control**	68 ± 3.55^*^^†^	62 ± 3.55^*^^†^	58 ± 3.55^*^^†^	59 ± 3.55^*^^†^	64 ± 3.55^*^^†^	62 ± 3.72^*^^†^	66 ± 3.72^*^^†^

*Parameters are reported as mean (± SE^c^; range) at 10-minute intervals*.

a *TG, treatment group*;

b *CG, control group*;

c *SE, standard error*;

d *sIBP, systolic invasive blood pressure; mIBP, mean invasive blood pressure; dIBP, diastolic invasive blood pressure; BPM, beats per minute*;

e *TP, timepoint*;

f * *Significantly different to KMMB at this timepoint (p < 0.05)*;

† *Significantly different to TZMB at this timepoint (p < 0.05)*;

g *na, not available: missing values / no measurements available*.

### Arterial Blood Gas Analysis

**Figure 3:**
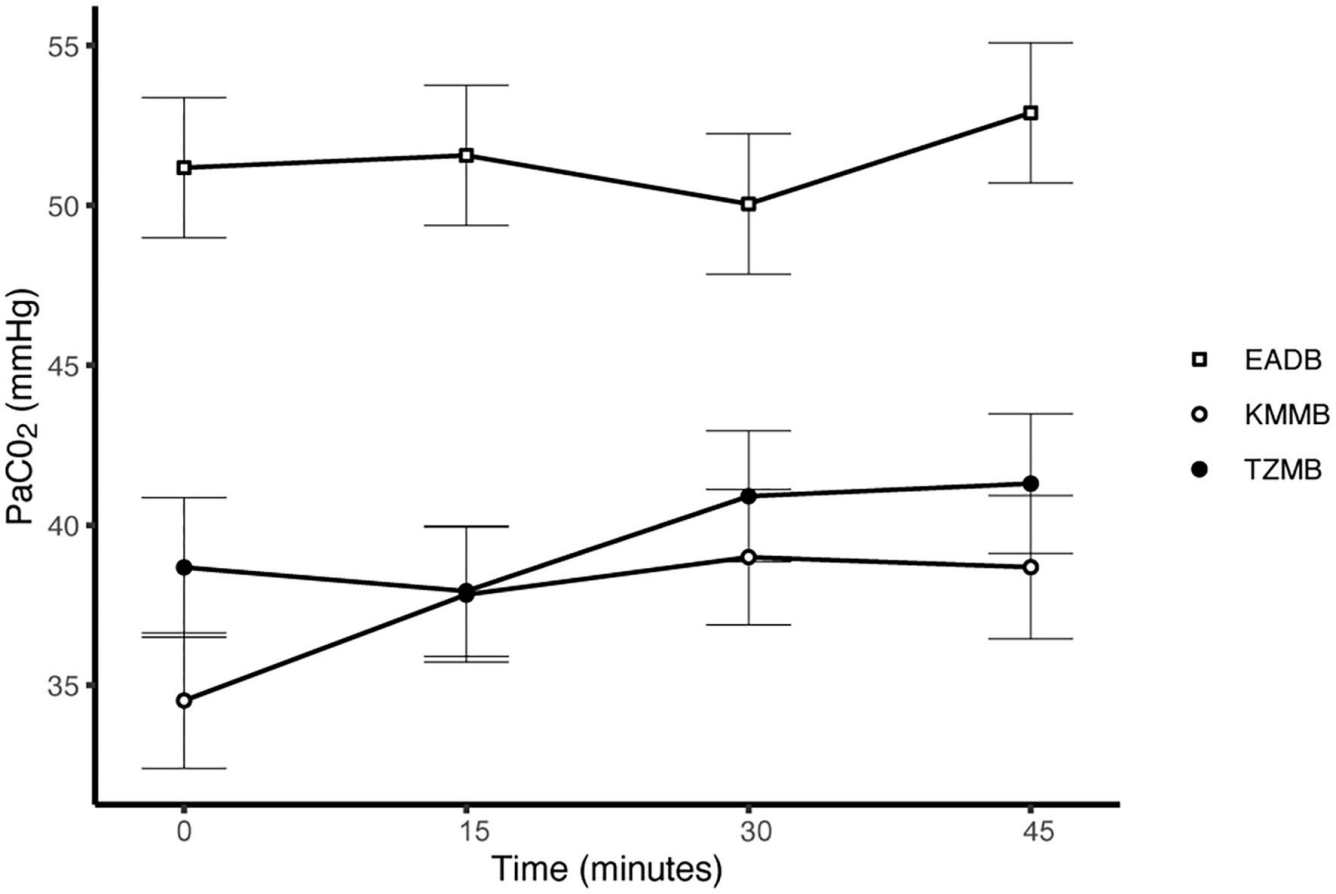
Illustration of partial arterial pressure of carbon dioxide (PaCO_2_) over time. Values at each timepoint are the means for treatment group 1 (KMMB), treatment group 2 (TZMB) and the control group (EADB) ± standard error (error bars).

**Figure 4:**
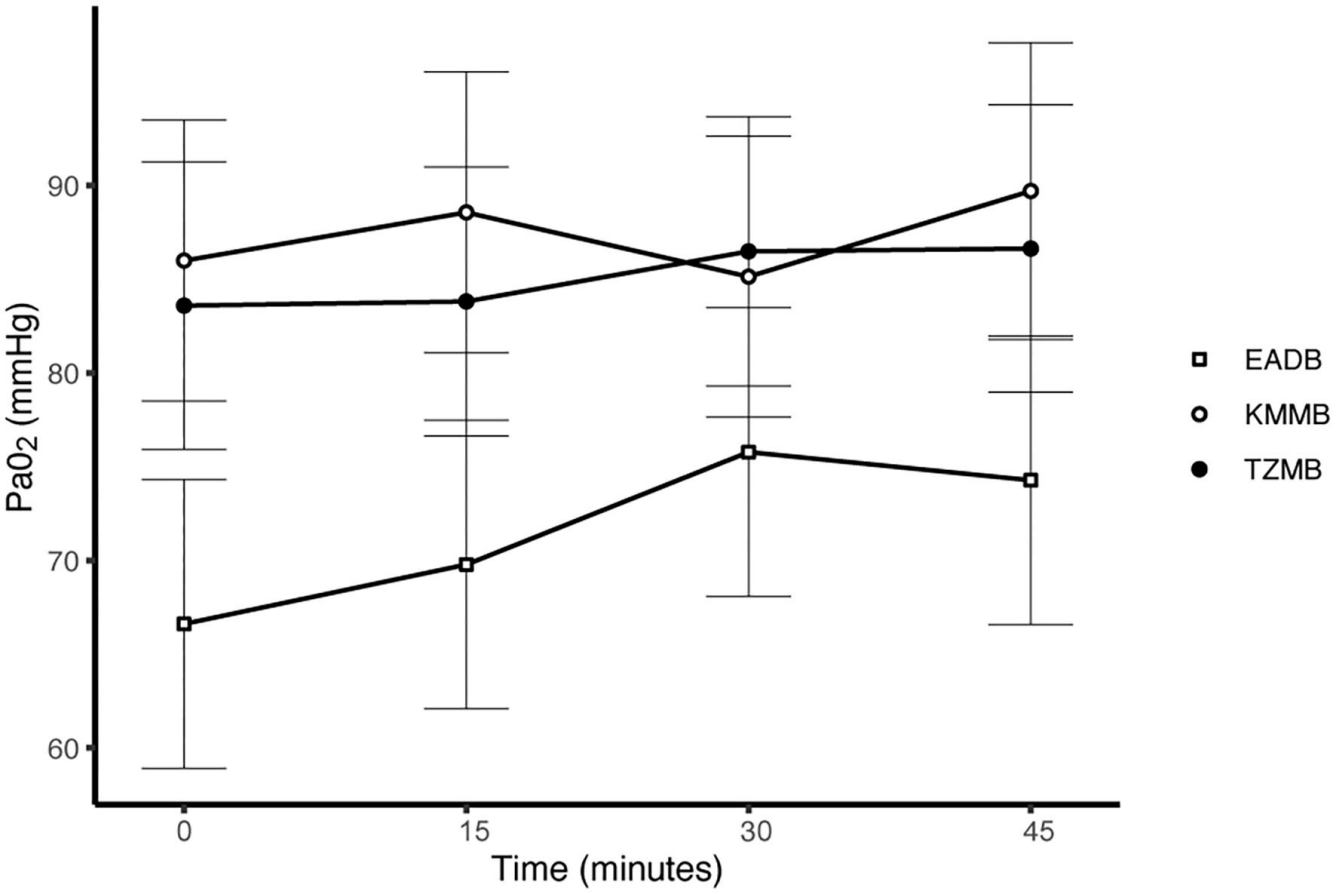
Illustration of partial arterial pressure of oxygen (PaO_2_) over time. Values of each timepoint are the means for treatment group 1 (KMMB), treatment group 2 (TZMB) and the control group (EADB) ± standard error (error bars).

**Figure 5:**
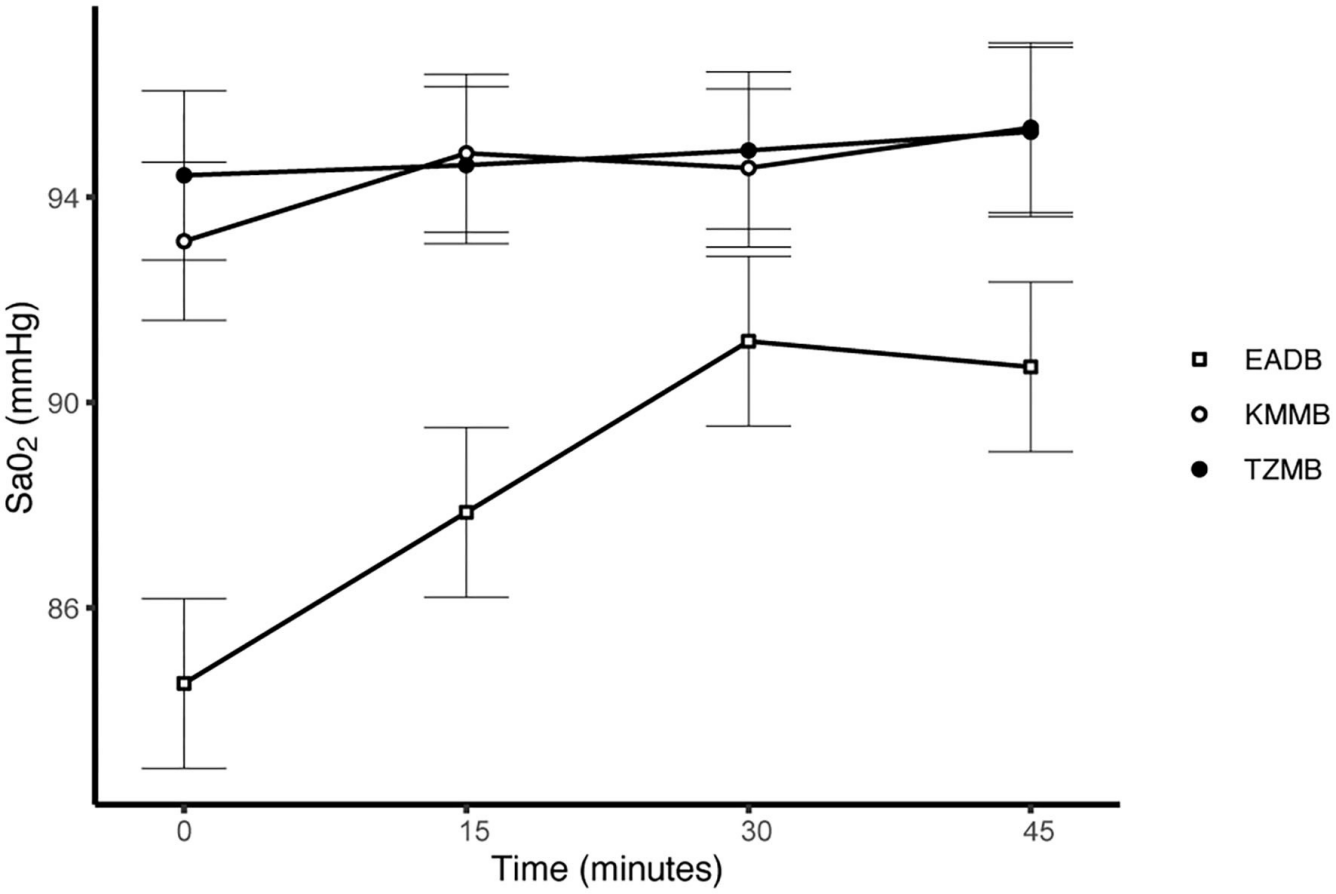
Illustration of oxygen saturation (SaO_2_) over time. Values of each timepoint are the means for treatment group 1 (KMMB), treatment group 2 (TZMB) and the control group (EADB) ± standard error (error bars).

PaCO_2_ levels indicated normocapnia for TG 1 and TG 2 compared to hypercapnia (PaCO_2_ > 45 mmHg) in the control group (Figure 3, Table 7). Hypoxemia (PaO_2_ < 80 mmHg) was found in the EADB kulans over the course of immobilization. However, hypoxemia was not detected in the treatment groups, even though oxygen was supplemented only after the first arterial sample was collected 10 min following recumbency (TP10) (Figure 4, Table 7). Before oxygen supplementation (TP 0), both treatment groups revealed higher PaO2 values and lower alveolar-to-arterial oxygen pressure gradients [P(A-a)O2] than the control group. Following oxygen supplementation, there was only a mild increase in PaO2 values, while P(A-a)O2 significantly increased in all three groups (Table 7). Moreover, both treatment groups exhibited significantly higher SaO_2_ values during the first 30 min of anesthesia than the hypoxemic control group (Figure 5, Table 7) (8).

**Table 7:** Blood gas parameters of TG^a^ 1: ketamine–medetomidine–midazolam–butorphanol (KMMB), TG 2: tiletamine–zolazepam–medetomidine–butorphanol (TZMB), and CG^b^: etorphine–acepromazine–detomidine–butorphanol (EADB), immobilized kulans (Equus hemionus kulan) following recumbency at timepoint 0 for a 45-minute period.

**Parameter^d^**	**Protocol**	**TP^e^ 0**	**TP 15**	**TP 30**	**TP 45**
**pH**	**TG 1**	7.35 ± 0.02	7.38 ± 0.02	7.41 ± 0.02	7.44 ± 0.02
	**TG 2**	7.37 ± 0.02	7.43 ± 0.02	7.43 ± 0.02	7.44 ± 0.02
	**Control**	7.29 ± 0.02^†^^f^	7.33 ± 0.02^†^	7.35 ± 0.02^†^	7.35 ± 0.02^†^^*^
**PaCO**_**2**_ **(mmHg)**	**TG 1**	34.5 ± 2.12	37.8 ± 2.12	39.0 ± 2.12	38.7 ± 2.24
	**TG 2**	38.7 ± 2.18	37.9 ± 2.04	40.9 ± 2.04	41.3 ± 2.18
	**Control**	51.2 ± 2.19^†^^*^	51.6 ± 2.19^†^^*^	50.0 ± 2.19^†^^*^	52.9 ± 2.19^*^^†^
**PaO**_**2**_ **(mmHg)**	**TG 1**	86.0 ± 7.50	88.6 ± 7.50	85.1 ± 7.50	89.7 ± 7.91
	**TG 2**	83.6 ± 7.67	83.8 ± 7.18	86.5 ± 7.18	86.6 ± 7.67
	**Control**	66.6 ± 7.70	69.8 ± 7.70	75.8 ± 7.70	74.3 ± 7.70
**BEecf (mmol/L)**	**TG 1**	−6.6 ± 1.50	−2.4 ± 1.50	0.1 ± 1.50	1.8 ± 1.52
	**TG 2**	−1.6 ± 1.28	1.7 ± 1.25	4.0 ± 1.25	4.7 ± 1.28
	**Control**	−2.9 ± 1.28	0.3 ± 1.28	1.6 ± 1.28^†^	2.8 ± 1.28
**HCO**_**3**_ **(mmol/L)**	**TG 1**	18.7 ± 1.25^†^	22.2 ± 1.23	24.4 ± 1.23	25.7 ± 1.25
	**TG 2**	23.1 ± 1.07	25.8 ± 1.04	27.8 ± 1.04	28.5 ± 1.07
	**Control**	23.1 ± 1.07^*^	25.7 ± 1.07	26.8 ± 1.07	28.1 ± 1.07
**TCO**_**2**_ **(mmol/L)**	**TG 1**	19.4 ± 1.24^†^	23.0 ± 1.24	25.4 ± 1.24	26.6 ± 1.26
	**TG 2**	24.0 ± 1.09	26.9 ± 1.06	28.9 ± 1.06	29.6 ± 1.09
	**Control**	24.5 ± 1.09^*^	27.0 ± 1.09	28.2 ± 1.09	29.7 ± 1.09
**SaO**_**2**_ **(%)**	**TG 1**	93.1 ± 1.54	94.9 ± 1.54	94.6 ± 1.54	95.4 ± 1.66
	**TG 2**	94.4 ± 1.65	94.6 ± 1.53	94.9 ± 1.53	95.3 ± 1.65
	**Control**	84.5 ± 1.65^†^^*^	87.9 ± 1.65^†^^*^	91.2 ± 1.65	90.7 ± 1.65
**Lactate (mmol/L)**	**TG 1**	6.20 ± 0.80	4.04 ± 0.80	3.08 ± 0.80	2.68 ± 0.82
	**TG 2**	4.44 ± 0.72	2.92 ± 0.70	2.47 ± 0.70	2.17 ± 0.72
	**Control**	6.99 ± 0.73	5.22 ± 0.73^†^	3.99 ± 0.72	3.40 ± 0.72

*Parameters are reported as mean (± SE^c^; range) at 15-minute intervals*.

a *TG, treatment group*;

b *CG, control group*;

c *SE, standard error*;

d *pH, arterial hydrogen ion concentration, negative logarithm; PaO_2_, partial arterial pressure of oxygen; PaCO_2_, partial arterial pressure of carbon dioxide; BEecf:, base excess; HCO_3_, arterial bicarbonate ion concentration; TCO_2_, total carbon dioxide; SaO_2_, arterial oxygen saturation*;

e *TP, timepoint*;

f * *Significantly different to KMMB at this timepoint (p < 0.05)*;

† *Significantly different to TZMB at this timepoint (p < 0.05)*.

## Discussion

Both treatment protocols effectively induced immobilization provided excellent muscle relaxation and a safe and reliable anesthesia. However, it should be highlighted that there were certain variations between the treatment groups and the control group, especially concerning side effects on the cardiorespiratory system. Evaluation of cardiovascular measurements showed elevated systolic arterial pressure in all three groups, although severe systemic hypertension (> 160 mmHg) was only observed in the kulans immobilized with TZMB (Table 6) (26). Assessment of respiratory function revealed normoxemia in the treatment groups, while the control group maintained hypoxemic over the course of immobilization (Figure 4, Table 7) (26). In addition, PaCO_2_ levels indicated normocapnia for the treatment groups, while the control group was hypercapnic (Figure 3).

The transition to recumbency in the kulans was characterized by a consistent pattern corresponding to increasing drug effects. The EADB kulans followed the typical course of etorphine inductions with a hypermetric “hackneyed” gait, stargazing, walking into fences, head pressing and tremors (7, 9, 11), while the treatment groups revealed similar characteristics as reported in previous studies on etorphine-free protocols in wild equids, such as standing with a wide based stance, head down, bottom lip hanging until stumbling and transition to recumbency (7, 13, 14, 19, 20). Inductions of the etorphine-free combinations were longer but smoother, while induction times of the EADB group were similar to the 5.6 min reported for the same dosages of this combination in free-ranging kulans (9). Due to the faster onset of the effect of ketamine, compared to tiletamine, induction times for the KMMB group were shorter than those for the TZMB group (26). Protocols using similar dosages of only ketamine and medetomidine in other equids resulted in longer inductions (13, 20). Due to the synergistic effects of adding butorphanol and midazolam, similar induction times with lower dosages of ketamine and medetomidine could be achieved (20). To the authors knowledge, this is the first comprehensive study incorporating the benzodiazepine midazolam into a total intramuscular anesthesia (TIMA) protocol of wild equids.

Both treatment protocols provided excellent muscle relaxation, while the current study, the control group revealed high EMG values, indicating a state of excitation without sufficient muscle relaxation. Etorphine-induced respiratory depression and thoracic muscular rigidity (31–35) may result in impaired alveolar ventilation. The consequences include respiratory acidosis, reflected in significantly higher PaCO_2_ and lower pH levels in the control group. Furthermore, etorphine-induced muscle tension, tremors and seizures (31–35) may result in increasing cellular metabolism, higher lactate levels and consequently a mild degree of metabolic acidosis. However, a greater degree of metabolic acidosis was detected in TG 1, while the high potency of the benzodiazepine zolazepam in TG 2 may have contributed to improved muscle relaxation and consequently lower lactate, a less negative base deficit and significantly higher bicarbonate levels (TP0 = *p* < 0.0325) (Table 7).

Increased muscle rigidity is a known side effect of etorphine, and severe seizures and continuing tremors were observed with several animals of the control group (26). The immobilization quality in the control group kulans was therefore scored as deep sedation. For the treatment groups, the qualitative assessment indicated a light to deep immobilization plane with maintained palpebral and anal reflexes. There was no evidence of spontaneous arousals as previously reported for Przewalski horses immobilized with medetomidine and ketamine (13). Both treatment groups did not require additional anesthetics until reversal after 60 min.

Systolic arterial hypertension combined with a presumably baroceptor-initiated lower pulse wave was detected in the treatment groups (7). TZMB kulans received a lower medetomidine dosage than KMMB kulans. However, severe systolic arterial hypertension was observed only in TZMB kulans, with a significantly higher systolic arterial pressure than both TG 1 and the control group. These differences indicate that the indirect cardiovascular impact of tiletamine in the TZMB group induces more pronounced hypertensive effects than ketamine in the KMMB group (7, 26). Systolic arterial pressure in both treatment groups was considerably lower than in zebras anesthetized with KMB (7). The addition of midazolam and the consequently have contributed to this improvement of cardiovascular parameters. Etorphine administration in horses enhances sympathetic activity and consequently produces tachycardia and an elevation of systemic blood pressure (33–35). However, in the current study, the EADB kulans showed only transient systemic hypertension decreasing over time to normotension, while sustained compensatory tachycardia was observed.

As a consequence of drug-induced hypoventilation, most immobilizations of wildlife are associated with hypercapnia (16, 36). PaO_2_ levels of < 80 mmHg indicate hypoxemia, while profound hypoxemia is of concern when values drop below 60 mmHg (26). PaCO_2_ levels in the treatment groups were comparable to awake equids (7, 26), while moderate hypercapnia was detected in the control group (Table 7). Both treatments showed normocapnia and a RR within normal limits. Etorphine reduces the sensitivity of arterial chemoreceptors, inhibiting respiratory response mechanisms to hypoxemia and hypercapnia (31). Hypoxemia (PaO_2_ < 80 mmHg) was initially observed only in the control group, where hypoventilation may have contributed to clinically significant hypoxemia and hypercapnia (26). However, although oxygen was administered from TP 10 onwards, hypoxemia did not resolve in the EADB group over the course of immobilization. Also the PaO_2_ levels only slightly increased following oxygen supplementation, regardless of the drug combination used (Figure 4). These findings indicate that other drug-induced mechanisms, apart from hypoventilation, may have contributed to the hypoxemia, such as elevated alveolar-arterial oxygen partial pressure gradients (P(A-a) O2) (26). Medetomidine and etorphine are known to cause pulmonary hypertension and consequently intrapulmonary shunting and V/Q-mismatching (36). Pulmonary hypertension leads to the formation of pulmonary congestion with interstitial oedema and decreases the capillary blood flow transit time. These mechanisms may contribute to hypoxemia by hindering gas exchange across alveolar capillary membranes (37). The findings of this study support these considerations with an elevated (P(A-a) O2) in all three groups compared to 10 mmHg reported for healthy horses at rest (Table 7).

Furthermore, medetomidine reduces peripheral oxygen availability, due to peripheral vasoconstriction (38). Observations in zebras immobilized with KMB appeared to be consistent with the above mentioned findings (7). Despite normocapnic PaCO_2_ levels, profound hypoxemia was observed in these zebras (7). These results indicate that the addition of midazolam has a dose-reducing effect on medetomidine, which may reduce pulmonary and peripheral hypertension (23).

The recovery times in the control group (EADB 3.5 ± 1.27 min) were significantly shorter than those in the treatment groups (KMMB [6 ± 1.10 min) and TZMB (6.29 ± 1.00 min)] and similar to the recovery times observed in free-ranging kulans with no evidence of re-narcotization (11). The kulans in both study groups had faster recovery times than ketamine-medetomidine immobilized feral donkeys (21 min) and Przewalski horses (13 min). As the administration routes of antagonists is similar, the differences in recovery times may be related to the higher atipamezole-to-medetomidine ratios used in kulans (5 mg/mg) compared to feral donkeys and Przewalski horses (2.5 mg/mg) (13, 20). It should also be taken into consideration that longer recumbency times in KMMB and TZMB kulans (60 min) may improve antagonist efficiency because the residual drug effects decrease over time. It has been reported in feral horses, that following comparable medetomidine dosages in a combination of medetomidine and ketamine, residual drug effects diminished, and horses recovered approximately 70 min after anesthesia induction, without any reversal of anesthetics (18). Recoveries of ketamine–medetomidine–butorphanol-immobilized zebras took 1.5 min to standing after atipamezole was administered (7.5 mg/mg medetomidine), partly intravenous and partly intramuscular (1:1) (7). KMB zebras and KMMB kulans received similar naltrexone dosages intravenously. The faster recovery times for these zebras may be related to higher atipamezole-to-medetomidine ratios (7.5 mg/mg), as well as to different intramuscular-to-intravenous ratios of atipamezole (7). The residual effects of midazolam may also be responsible for the longer recovery times in KMMB kulans. The flumazenil-to-midazolam ratio (0.005 mg/mg) used in this study was fairly low; therefore, further studies should be conducted to determine the influence of residual midazolam effects on recovery time and quality.

Tiletamine–zolazepam–medetomidine-immobilized feral horses needed 31 min to recover from 60 min of recumbency (17). Their immobilization times were comparable to the kulans, but the differing atipamezole-to-medetomidine ratios for feral horses (0.7 mg/mg) may also be responsible for the longer recovery times (17). In conclusion, higher atipamezole-to-medetomidine and flumazenil-to-midazolam ratios may be beneficial, especially for shorter procedures, as the main anesthetic effects are still present.

The recovery qualities for the different study groups were excellent, regardless of the drug combination used. Reports on the immobilization of free-ranging wild equids emphasize preventing rapid attempts to stand up immediately after the administration of antidotes. The aim of these measures is to coordinate the first attempt to stand with sufficient antagonist effects. Physical restraint in a lateral position with fixation of the head was therefore recommended, to facilitate rapid and safe recoveries (11). In a study on feral horses, sternal positioning was mentioned to have beneficial effects on recovery quality (18). The sporadic pre-study use of the KMMB combination (unpublished data) revealed that sternal positioning without any physical restraint may improve the recovery qualities of kulans. This technique was applied in our study and it resulted in controlled, calm, and smooth recoveries and hence reduced stress for the animals. Therefore, we recommend sternal positioning during recovery for other captive equine species anesthetized with the KMMB and TZMB protocol.

In conclusion, the essential benefit of the KMMB and TZMB protocol is an excellent muscle relaxation. Both combinations provided effective and safe inductions, smooth recoveries, and an adequate and reliable depth of anesthesia suitable for prolonged procedures on captive kulans.

Although species-specific differences require further evaluation, the findings in this study indicate that supplementing KMB with midazolam provides excellent muscle relaxation and considerably improves the cardiorespiratory parameters. With regard to the promising results of the non-opioid-based KMMB combination of the current study, this protocol should be further investigated in other wild equids.

## Data Availability Statement

The raw data supporting the conclusions of this article will be made available by the authors, without undue reservation.

## Ethics Statement

The animal study was reviewed and approved by the Committee for Ethics and Animal welfare of the Leibniz-IZW.

## Author Contributions

JB, JP, and FG designed the study protocol, performed anesthesias, and carried out the clinical examinations. DB and EG assisted with clinical examinations. DB performed laboratory diagnostics. HR completed the statistics. JB drafted the manuscript. BE added comments and reviewed blood pressure results. JP, AH, FG, EG, HR, BE, and DB conducted review and manuscript conceptualization. All authors read and approved the final manuscript version.

## Funding

The publication of this article was funded by the Deutsche Forschungsgemeinschaft (DFG, German Research Foundation) – Projektnummer 491292795.

## Conflict of Interest

The authors declare that the research was conducted in the absence of any commercial or financial relationships that could be construed as a potential conflict of interest.

## Publisher's Note

All claims expressed in this article are solely those of the authors and do not necessarily represent those of their affiliated organizations, or those of the publisher, the editors and the reviewers. Any product that may be evaluated in this article, or claim that may be made by its manufacturer, is not guaranteed or endorsed by the publisher.
